# Colonization of malaria vectors under semi-field conditions as a strategy for maintaining genetic and phenotypic similarity with wild populations

**DOI:** 10.1186/s12936-014-0523-0

**Published:** 2015-01-21

**Authors:** Kija R Ng’habi, Yoosook Lee, Bart G J Knols, Dickson Mwasheshi, Gregory C Lanzaro, Heather M Ferguson

**Affiliations:** Ifakara Health Institute, Environmental Health and Ecological Sciences Thematic Group, Ifakara, Kilombero, Morogoro United Republic of Tanzania; Vector Genetics Laboratory, Department of Pathology, Microbiology and Immunology, School of Veterinary Medicine, University of Calfornia, Davis, USA; In2Care BV, Costerweg 5, 6702 AA Wageningen, The Netherlands; Institute of Biodiversity, Animal Health & Comparative Medicine, College of Medical, Veterinary & Life Sciences, University of Glasgow, Glasgow, G12 8QQ UK

**Keywords:** Malaria, Insect vectors, Semi field system, Fitness, Colonization

## Abstract

**Background:**

Malaria still accounts for an estimated 207 million cases and 627,000 deaths worldwide each year. One proposed approach to complement existing malaria control methods is the release of genetically-modified (GM) and/or sterile male mosquitoes. As opposed to laboratory colonization, this requires realistic semi field systems to produce males that can compete for females in nature. This study investigated whether the establishment of a colony of the vector *Anopheles arabiensis* under more natural semi-field conditions can maintain higher levels of genetic diversity than achieved by laboratory colonization using traditional methods.

**Methods:**

Wild females of the African malaria vector *An. arabiensis* were collected from a village in southern Tanzania and used to establish new colonies under different conditions at the Ifakara Health Institute. Levels of genetic diversity and inbreeding were monitored in colonies of *An. arabiensis* that were simultaneously established in small cage colonies in the SFS and in a large semi-field (SFS) cage and compared with that observed in the original founder population. Phenotypic traits that determine their fitness (body size and energetic reserves) were measured at 10^th^ generation and compared to founder wild population.

**Results:**

In contrast to small cage colonies, the SFS population of *An. arabiensis* exhibited a higher degree of similarity to the founding field population through time in several ways: (i) the SFS colony maintained a significantly higher level of genetic variation than small cage colonies, (ii) the SFS colony had a lower degree of inbreeding than small cage colonies, and (iii) the mean and range of mosquito body size in the SFS colony was closer to that of the founding wild population than that of small cage colonies. Small cage colonies had significantly lower lipids and higher glycogen abundances than SFS and wild population.

**Conclusions:**

Colonization of *An. arabiensis* under semi-field conditions was associated with the retention of a higher degree of genetic diversity, reduced inbreeding and greater phenotypic similarity to the founding wild population than observed in small cage colonies. Thus, mosquitoes from such semi-field populations are expected to provide more realistic representation of mosquito ecology and physiology than those from small cage colonies.

## Background

Despite intensified control efforts, malaria still account for an estimated 207 million cases and 627,000 deaths worldwide each year [1]. However malaria mortality rates have fallen by 45% globally since 2000, and by 49% in the sub-Saharan Africa alone [1]. To build on these achievements and generate further reductions, it is likely that novel, alternative means of vector control will be needed to complement traditional front line methods, such as insecticide-treated nets, indoor residual spraying, and drugs [2-4]. Of potential alternatives, one that is highly dependent on the use of realistic, semi-field systems is the potential use of genetically-modified (GM) and/or sterile male mosquitoes into natural populations, where they would mate successfully with wild females and introduce the desired traits [5,6]. This is because the successful implementation of any GM strategy is dependent on the fitness of modified individuals in nature, which must be evaluated under realistic, but contained conditions before releases are made [7]. The need to develop a colonization system that would maintain the natural phenotypic and genetic characteristics of mosquito vectors destined for research and release under such programmes fuelled the enthusiasm to undertake this study.

The laboratory colonization of malaria vectors is intended to provide valuable insights into the biology of their corresponding wild population. However, use of colonies for comprehensive investigation of mosquito vector biology may have limited application because of several constraints including: (1) some species/subspecies may be difficult to colonize [8,9], (2) mating patterns and the fitness of hybrids under colony conditions may be different from natural settings [10-13], and/or (3) the susceptibility of mosquitoes to parasite infection or insecticides may be different from natural populations [14-17]. These effects are hypothesized to arise because the genetic composition of colonized vectors often deviates from their original founder population. This is evidenced by the representation of fewer alleles and lower mean heterozygosity [18-20] in laboratory colonies, even when such colonies are maintained at relatively large population sizes (i.e. ≥ 5,000 individuals) [21]. Such changes in the genetic composition of laboratory colonized populations are often accompanied by the appearance of undesired and/or unrealistic phenotypic and behavioural traits, such as poor mating ability [22-24], a phenomenon that could be either a cause or consequence of reductions in genetic diversity [25]. Also laboratory populations in some *Anopheles* colonies are reported to have high immune responses compared to their counterpart, field populations [26,27]. Thus it remains unclear how realistically studies of vector competence, ecology and behaviour conducted on laboratory colonies can accurately describe their wild counterparts.

Despite these limitations, laboratory colonies remain an invaluable asset to research on mosquito vectors, by providing a stable source of standardized material, which is necessary for high throughput experimental study and detailed hypothesis testing under controlled settings. Thus the major need is to produce mosquitoes under colony conditions that are more behaviourally and genetically representative of their field counterparts. Experimental studies using colonized mosquito vectors could play a vital role in elucidating aspects of their biology and susceptibility to control measures that may be otherwise intractable to measure under field conditions [15,28,29]. Thus the need for an improved insect colonization process that avoids such detrimental effects and maintains realistic level of genetic variation is crucial.

Contained semi-field systems (SFS) [30-32] have been proposed as an intermediate strategy for moving laboratory-based research into full field application [31,33]. Semi-field systems are large enclosures, established within the natural environment of target mosquito vectors, containing habitat features necessary for the completion of mosquitoes life cycle. This contrasts with conditions of standard laboratory colonies under which mosquitoes are kept in small cages, exposed to fixed climatic and light conditions, with access to limited, standardized food. Mosquitoes within SFS are exposed to more realistic range of environmental heterogeneity including varying ambient climatic conditions [30,31,34], can access a wider range of microhabitats (e.g. resting sites) and dietary resources (e.g. plant sugar sources) than those in standard laboratory colonies. Unlike in laboratory colonies, mosquitoes in SFS can fly freely over distances of several meters to select mates, blood meal sources (often provided by the presence of an entire live host), and larval habitats from the range of microhabitats that are available. The greater ecological diversity of SFS may also support a wider range of genotypes than can persist in laboratory colonies, where strong selection for individuals with particular characteristics can occur. Finally, the larger size of SFS compared to standard laboratory cages (even when many are used) means that a larger total population size of individuals can be accommodated, which in itself could be sufficient to maintain higher levels of diversity and reduce inbreeding. On this basis, it is hypothesized that mosquitoes colonized in SFS could retain a substantially higher degree of genetic and phenotypic similarity to their field counterparts through time than those reared in standard laboratory colonies. This prediction however has not yet been tested probably due to the fact that until recently there were relatively few semi-field systems available for comparative analysis. Despite several prospective advantages of semi field systems [33-35], to date very few vector populations have been successfully established in such conditions [31], with only one population being reported to have been established for several generations [36] as would be required to observe genetic changes over time. Demonstration that SFS colonies can produce individuals that are more representative of natural populations than existing methods, would strengthen the case for the expanded use of these facilities.

This study presents the first analysis of the long-term population genetic dynamics of *Anopheles arabiensis*, one of the most important vectors of human malaria in Africa [35,37-39], after colonization under semi-field conditions. This study was conducted in an area of southern Tanzania with high malaria transmission intensity which is largely due to *An. arabiensis* [40,41]. As has been described elsewhere [36], a self-replicating population of *An. arabiensis* was established within a large-scale semi-field system at the Ifakara Health Institute in 2008. Simultaneously with establishment of the SFS colony, individuals from the same founding population in Sagamaganga were used to establish two additional colonies (2 separate lines) that were maintained in small cages typically used in standard laboratory colonies. Over a period of multiple generations, the genetic diversity of mosquitoes in all colonies was repeatedly monitored and contrasted with that of their original founding wild population. In addition to assessing genetic variation, regular measurements of mosquito body size and the abundance of their energetic reserves were also taken. As variation in these traits are known to be a primary determinant of numerous mosquito life-history traits including survival and reproduction [23,24,42], and can predict variation in mosquito population dynamics [43], were used as a proxy to assess mosquito fitness under different colonization conditions. Key aims of this study were both to test the fundamental prediction that populations maintained under more environmentally heterogeneous conditions retain higher genetic diversity [44] and more specifically to demonstrate whether mosquito vectors colonized under semi-field conditions can provide a more realistic representation of wild type individuals than those obtained from small cage colonies.

## Methods

Recently blood fed, female mosquitoes that were visually identified as belonging to the *Anopheles gambiae s.l.* species complex were collected from 16 houses and five livestock sheds in Sagamaganga village (Southern Tanzania, 8.0667 S; 36.8000 E) using mouth aspirators in May 2008. Collections were conducted at households and all collected *An. gambiae s.l.* females were transferred into a holding cage (15 cm^3^) and taken to the Ifakara Health Institute (IHI) where they were held for 1 day before being transferred into individual Styrofoam cups (4.5 × 7 × 7.5 cm) for oviposition. After egg laying (3–5 days), all females were killed and subjected to PCR analysis to confirm their species [30]. Eggs of all of those females identified as *Anopheles arabiensis* were pooled and randomly allocated for use to use to either establish a new semi-field colony, or one of two small cage colonies as described below (and in Table 1).

**Table 1:** **Comparison of the maintenance conditions used in the small cage and semi-field colonies of** ***An. arabiensis*** **studied here**

**Description**	**Small cage colony**	**Semi-field colony**
Founding individuals	1,800/line	~3,000
Containment size	35 × 35 × 35 cm	21 × 9.1 × 7.1 m
Environmental control	Ambient	Ambient
Larval food	Tetramin	Natural (microbial growth)
Adult food	Glucose solution (10%)	Natural (plant sugars)
Blood meal source	Human	Cow

### Small cage colony establishment and sample collection

*Anopheles arabiensis* small cage colonies were maintained in an insectary within a large enclosed SFS [30], in which temperature and humidity were not controlled and varied depending on the ambient conditions. Here two lines of *An. arabiensis* were established within a series of small, environmentally homogeneous laboratory cages (35 cm^3^) typically used in most laboratory colonies. These small cage colonies were established by gradual release of first filial generation (F1) larvae from field collections into in plastic rearing trays (diameter 43 cm; water depth 5–5.5 cm) and maintained on fish food (Tetramin™). Approximately 1,800 first instar larvae (F1) per line were used to establish each small cage colony, being introduced over a one-week period from field collected females (~700). Pupae developing in these rearing trays were moved into a series of small cages (35 cm^3^) which and maintained on an *ad libitum* diet of 10% sugar solution for the maintenance of adult females and males mosquitoes.

Within these small cage colonies, the interior of cages was bare except for the presence of small jar with a 5% sugar solution consumed through filter paper and the small bowls used to introducing pupae from rearing trays. Female mosquitoes were provided with blood meals by feeding on a human arm for 10 min, 3 times per week. Three day after each blood feed, a small bowl containing water was put into the cage overnight to provide a substrate for mosquitoes to lay their eggs on. Generations were separated by moving all “new eggs” produced into separate rearing trays and transferring the resultant pupae into new adult cages for emergence. All rearing trays and cages were labeled on the basis of their generation number relative to the original generation that began the colony. For each generation, a subsample of 100 individuals (50 males + 50 females) were randomly collected from each adult cage using a mouth aspirator and stored individually in tubes containing silica gel for molecular analysis. Samples were collected for over ten generations. A subsample of 30 adult males were collected at the 10^th^ generation and used for assessing body size differences and energetic reserve abundance.

### Semi-field system colony establishment and sample collection

**Figure 1:**
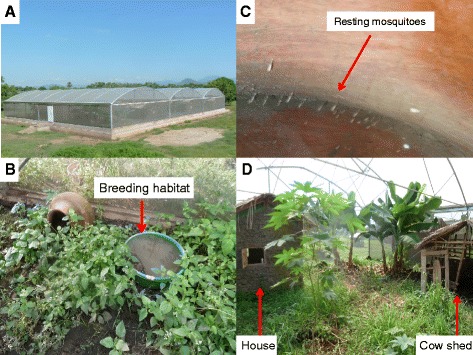
**The IHI semi field system in which small cage colonies and the large cage SFS colony was established. (A)** outside view of the semi field system; and within the large semi-field cage: **(B)** a breeding habitat **(C)** mosquitoes resting in a clay pot and **(D)** the interior of the semi-field cage including a replica of a local house, cow shed and typical vegetation.

A contained semi-natural environment of 21 × 9.1 × 7.1 m was created within a 700 m^2^*Anopheles* mosquito SFS built at the IHI in southern Tanzania [30] (Figure 1A). The inside of this chamber was designed to mimic the natural habitat of *Anopheles* mosquitoes. Habitat features included vegetation that emerged from seeds that were introduced into the compartment with the soil layer. Other plants that are common in natural habitats, such as plantain banana (*Musa paradisiacal*), castor bean (*Ricinus communis*) and sweet potato (*Ipomoea batata*) were also planted [36]. Artificial larval habitats were constructed using plastic buckets (43 cm diameter, Figure 1B). These were partially filled with soil to allow microbial and/or algal growth. Approximately 3,000 *An. arabiensis* larvae, obtained from field-collected females (~600) were released into these larval habitats over a one-week period in May 2008. The conditions in the SFS (temperature and humidity) varied depending on the ambient conditions. Larvae were not provided with any additional food other than algae and microorganisms naturally growing within artificial habitats. Adults emerging from these habitats were allowed to freely move and rest throughout the SFS compartment (Figure 1C). Adult females were given the opportunity to obtain blood meal from a calf placed in the cattle pen inside the SFS seven (7) nights per week (Figure 1D).

In contrast to the cage colonies, the SFS population allowed for generations to overlap, such that at any given sampling point, the precise number of generations from which each individual had passed since founding could not be precisely confirmed. However, a good estimate of the approximate generation number of individuals sampled at different time points was estimated on the basis of previous knowledge of *An. arabiensis* generation length under these semi-field conditions [36]. Previously, it was observed that the time difference between one generation under ambient SFS conditions is approximately 25 days (21–25 days) [36]. Applying this guideline, a total of 100 emerging adults (50 males + 50 females) were sampled from larval habitats using emergence traps over a period of 5 days at an interval of 21–25 days to approximate different generations. This process was repeated every generation from the 1^st^ to 10^th^ generation with individuals collected in tubes with silica gel for subsequent genetic analysis. As with the small cage colonies, a subsample of male mosquitoes was collected at the 10^th^ generation and used to assess body size differences and energetic reserve abundance with respect to the wild population. For these comparisons, new field samples were also collected from the original founder population at the same time (e.g. approximately 10 months later after the initial establishment of the SFS and cage colonies. Samples were collected from the same houses and livestock sheds as in the original collections. A subset of fifty male mosquitoes was stored in tubes with silica gel for further genetic analysis.

### Phenotypic analysis

Sub samples of males from small cage and SFS colonies (10^th^ generation) were used to compare differences in energetic reserve abundance between cage and SFS colonies. Samples were also collected from the founding population of semi field system and cage colonies. Collection was done in the morning and all collected males were anaesthetized immediately after collection. Individuals were then put into individual glass tubes, crushed and analysed for lipids, glycogen and glucose [45]. Wing size has traditionally been used as a good proxy of body size in mosquitoes. Thus, a sub sample of males from cage colony, SFS colony and wild population were collected from the 10^th^ generation and used for wing length measurement. From each individual one wing was removed and measured under dissecting microscope.

### Microsatellite DNA analysis

In this study, only males from generations 1, 2, 5 and 10 from small cage and SFS colonies were used for genetic analysis. Females were not used in order to preserve them for future breeding. To estimate the relative degree of genetic diversity in the different colonies, 50 males from these generations were screened for 11 microsatellite loci from chromosome 2 and 3 [46-48]. The 11 microsatellite markers used were as follows: *AG2H175*, *AG2H85*, *AG2H164*, *AG2H197* and *AG2H675* on chromosome 2; and *AG3H127*, *AG3H249*, *AG3H812*, *AG3H311*, *AG3H811* and *AG3H93* on chromosome *3*. Microsatellite loci were PCR-amplified from individual mosquito DNA, using flanking primers that have been previous described [48,49]. Each 11 μl PCR reaction consisted of 5 μl of Multiplex master mix, 1 μl primer mix and 4 μl of Rnase free water. The primer mix was made to a final volume of 250 μl, consisting of varying amounts (μl) of each primer and variable amount of TE buffer depending on the number and amount of primers mixed. The forward primer in each reaction was labeled with a fluorescent marker (FAM, NED or HEX) compatible with ABI PRISM® 3130 Genetic Analyzer (Applied Biosystems). DNA amplifications were completed in MJ Research PTC-200 thermal cyclers (MJ Research, Watertown, MA). A 5 min denaturation step at 95°C, followed by 29 cycles of 20 s at 95°C, 30 s at 55°C and 30 s at 72°C. A final extension at 72°C was extended to 1 hr to alleviate problems associated with addition of non-template nucleotide (dA) to the PCR products. 0.5 μl of PCR products was diluted in 20 μl of deionized H_2_O before it was mixed with 0.5 μl of GeneScan (GeneScan™ 400HD ROX™, Applied Biosystems), size standard and 12 μl of Hi-Di formamide. Mixtures were denatured in MJ Research PTC-200 thermal cyclers (MJ Research, Watertown, MA), for 5 min at 95°C, before being run on an ABI 3130 Genetic analyzer. Output was analysed using ABI PRISM® 3130 Genemapper (Applied Biosystems).

### Data analysis

Microsatellite polymorphism was compared between laboratory, semi-field and field populations. A PopIworkbench was used to store and manage the data [50]. For Arlequin compatibility, data format was converted using the tool provided by PopI workbench. The polymorphism was evaluated by comparing estimates of mean heterozygosity and the number of alleles at each of the 11 loci from chromosome 2 and 3. The Arlequin (ver. 3.11) programme was used to calculate the allele frequency and observed heterozygosity for each locus in each population. The t test was used to compare the differences between SFS and small cage colonies mean observed heterozygosity. Linear regression analysis was used to examine variation in the mean observed heterozygosity through increasing generations of colonization. Also, analysis of molecular variance (AMOVA) was used to estimate degree of inbreeding depression for all colonies. 10,100 permutations were performed to test for significance. The genetic distance between populations (F_*ST*_) was calculated and estimated the degree of inbreeding (F_*IS*_). General Linear Models were used to test whether the phenotypic traits of wing length, and energetic reserves (lipids, glucose and glycogen) varied significantly between colonies using SPSS software. A turkey HSD test was used for multiple comparisons.

## Results

**Figure 2:**
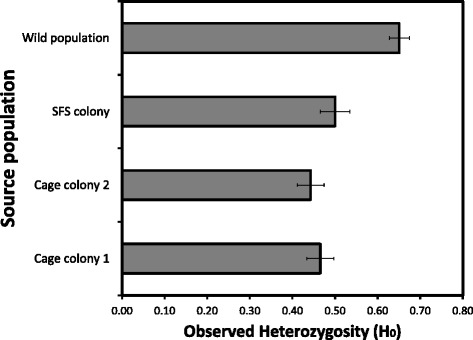
**Mean observed heterozygosity of field, semi field and small cage colonies for 11 microsatellite loci over ten generations.** Bars represent one standard error.

A total of 602 mosquitoes pooled from all 1, 2, 5 and 10 generation were used for microsatellite analysis from different mosquito colonies (Table 2). Microsatellite analyses indicated that the SFS colony had an overall observed heterozygosity (H_O_) of 49.8% (range 48-50%), which was higher than the overall observed heterozygosity of cage colony 1 (H_o_ = 47.3%; range 46.7-48.5%) and for cage colony 2 (H_o_ = 46.5%; range 45.2-47.4%). These differences in the H_o_ with the SFS colony were estimated to be statistically significant for both cage colony 1 (t = 9.41, df =2, p =0.01) and that of cage colony 2 (t =11.4, df =2, p < 0.001, Figure 2). The H_o_ of the two cage colonies were not significantly different from one another (t =1.1, df =1, *p* = 0.31). The observed heterozygosity of the founding wild population was significantly higher (H_o_ = 65%) than SFS colony (t = 40.7, df = 1, p < 0.001), cage colony 1, (t =30.6, df = 1, p < 0.001) and cage colony 2 (t =34.1, df =1, p < 0.001).

**Table 2:** **Number of samples of** ***An. arabiensis*** **males for which DNA was successfully amplified for microsatellite analysis in different colonies and generations in this study**

Estimated Generation*	**Wild population**	**Cage colony 1**	**Cage colony 2**	**SFS colony**
1		49	48	47
2		48	48	47
5		47	46	48
10	50	48	48	47
Total amplified	50	192	190	189

*Indicates the estimated number of generations since these colony populations were taken from their wild field population.

**Figure 3:**
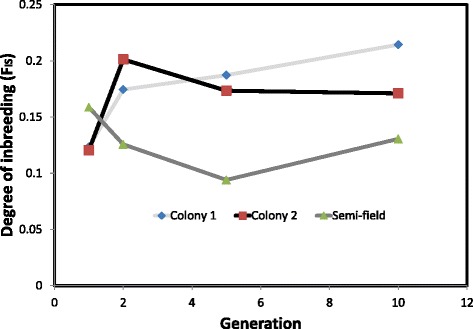
**The observed degree of inbreeding (** ***F***_IS_ **) in the small cage and semi field colonies over 10 generations.**

Analysis of molecular variance (AMOVA) for all generation (pooled) in small cage and SFS colonies indicated that, the major source of variation observed in all populations was between rather than within individuals; with inter individual variation accounting for 80% of the total observed genetic variation. The estimated degree of inbreeding in the SFS colony (F_*IS*_ = 0.10353) was significantly lower than in small cage colony 1 (W = 4, p = 0.03), small cage colony 2 (W = 0, p = 0.03) and small cage colonies combined (W = 28, *p* = 0.02) which decreased with increasing number of generations (Figure 3). Based on the *F*_ST_ estimates, while the SFS colony was genetically closer to the wild population (*F*_ST_ = 0.078), the cage colonies were genetically distant to the field population (*F*_ST_ = 0.167). The SFS colony was intermediate between wild population and cage colonies.

**Figure 4:**
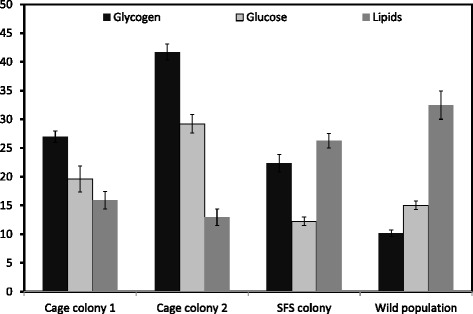
**The mean abundance of three mosquito energetic reserves (glucose, glycogen and lipids) in male** ***An. arabiensis*** **from small cage colony, SFS colony and the wild founder population.** Estimates are taken from the 10^th^ generation that had passed since the founding of the colonies. Error bars represent one standard error.

A total of 180 males (cage colony 1, N = 30, cage colony 2, N = 30, SFS colony, N = 90, wild population N = 30) only from 10^th^ generation, were used for energetic reserve analysis. There were significant differences between the abundance of energetic reserves in male *An. arabiensis* between cage colonies, SFS colony and the founding wild population (F_3, 176_ = 22.315, p <0.001). Males from SFS colony had higher abundance of lipids than both cage colony 1 (F_1, 118_ = 19.77, p < 0.001) and cage colony 2 (F_1, 117_ = 32.15, p <0.001). Similarly, wild population had higher abundance of lipids than small cage colony 1 (F_1, 58_ = 32.83, p < 0.001) and cage colony 2(F_1, 58_ = 47.07, p < 0.001). However, wild population had significantly lower levels of glucose than cage colony 1 (F_1, 58_ = 62.85, p < 0.001) and cage colony 2 (F_1, 58_ = 166.56, p < 0.001). SFS colony had significantly lower abundance of glucose than cage colony 1 (F_1, 117_ = 108.55, p < 0.001) and cage colony 2 (F_1, 118_ = 16.35, p < 0.001, Figure 4).

**Figure 5:**
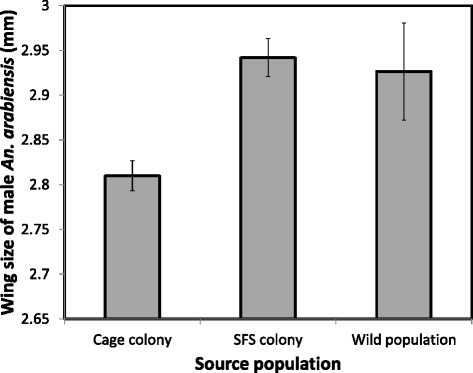
**The mean wing length of a subsample of male** ***An. arabiensis*** **from all colonies and the wild founding population, estimated at the 10** ^th^ **generation from when the colonies were founded.** Error bars represent one standard error.

The body size of male *An. arabiensis* maintained in the SFS colony was not significantly different from that measured in the wild founding population (*F*_1, 181_ = 0.80, *P =* 0.78). However, males maintained in the small cage colonies were significantly smaller in size than both those from the SFS colony (*F*_1, 198_ *=* 27.26, *P <* 0.001) and the wild population (*F*_1, 181_ = 5.72, p = 0.02, Figure 5). Furthermore, males from the small cage colonies (pooled across lines) showed a smaller range of variation in body size (range: 2.40 -3.20 mm) than that observed in those from the SFS colony (range: 2.30 – 3.30 mm) and the field population (range: 2.21 – 3.67 mm).

## Discussion

This study shows that *Anopheles* mosquito vectors maintained in a large cage semi-field colony provide a more realistic representation of those living in wild populations than do those produced from traditional insectary colonies where mosquitoes are maintained in small, environmentally homogeneous cages. The primary differences between mosquito vectors reared in small cage and the semi-field colony is that the latter retained a higher degree of genetic diversity through time after colonization, and closer phenotypic similarity to the originating field population. There are five sources of evidence to support these conclusions: (1) the mean observed heterozygosity (H_o_) in the SFS population of *An. arabiensis* was significantly higher than in either of the two small cage colonies that were founded from the same wild population; (2) the degree of genetic divergence (F_st_) between the SFS colony and the wild founder population was approximately half that as observed in small cage colonies; (3) the amount of inbreeding (F_IS_) observed over ten generations of colonization was significantly higher in small cage colonies than in the SFS colony; (4) the mean abundance of energetic reserves and their pattern of allocation was relatively similar in wild mosquitoes and those reared in the SFS colony, but significantly altered in mosquitoes reared in small cage colonies; and finally (5) the mean and range of male *An. arabiensis* body sizes were similar in the wild population and SFS colony, but significantly reduced in males from the small cage colonies. Therefore, colonizing and maintaining malaria mosquito vectors within a large semi field system can provide substantial advantages over typical, small cage colonies by yielding mosquitoes that are much more genetically and phenotypically similar to the wild populations which they are intended to represent.

The mean observed heterozygosity of both the wild and SFS *An. arabiensis* populations in this study are within the range of values that have been estimated using microsatellite analysis of a variety of wild African *Anopheles* vector populations (e.g. 0.48–0.89; [18,19]). Although the mean observed heterozygosity found in the smaller cage colonies in this study was lower than in the wild or SFS population, these values were higher than has been reported in small scale laboratory colonies of other insect species including sandflies [20], *Drosophila* [51,52] and *An. gambiae s.s.* [51,53]. This relatively enhanced heterozygosity within the small cage colonies may be due to the fact that unlike in many other traditional laboratory insect colonies, these mosquito lines were maintained under variable ambient conditions of natural light, temperature and humidity. Further study is needed to assess whether this apparently higher heterozygosity in these small cage colonies can be maintained over a long time period, but on the basis of results so far the authors hypothesize that the exposure of insects to more variable environmental conditions in a colony may help promote the maintenance of genetic variation [44].

There are several potential explanations for why the SFS colony of *An. arabiensis* maintained a higher degree of genetic variation and was more phenotypically similar to its wild founder population than males produced from small cage colonies. Firstly, within the SFS mosquitoes were allowed to freely fly from one location to another over a distance of several meters; providing them with an opportunity to experience varying environmental conditions and respond to cues related to hosts, predators, mates and oviposition sites that are similar to those in wild populations. This range of environmental conditions may have in itself supported a wider range of genotypes, as it does not impose the same intense, hard selection for the very limited range of behaviour that are necessary for reproduction within the confines of a small cage (e.g. ability to blood feed artificially, mate in a small area). Secondly, within the SFS both immature and adult mosquitoes obtained food resources of a similar source and manner as they would be consumed in the wild. Specifically, larvae fed on microbiota that developed naturally in water pools, adult males on natural plant sugar sources, and adult females from a live calf that spent a night within the system. Unlike the small cage colonies, no type of artificial food supplement was added to the SFS (e.g. fish food for larvae, glucose solution for adults), and the different nutritional values of these natural and artificial food sources may have been responsible for skewing the pattern of energetic reserve allocation (storage of lipids, sugars and glycogens) between small cage colonies and the SFS and wild population. The provision of natural food sources and more realistic environmental cues within the SFS may have further allowed mosquitoes to express genes (e.g. for host seeking and mate searching) that may be rendered unnecessary for fitness and thus likely to be lost within the more artificial confines of small cages. Theories on how environmental heterogeneity can maintain or promote genetic diversity are based on at least two potential, non-exclusive mechanisms: (i) environmental heterogeneity directly promotes diversity by providing a variety of different selective forces that potentially trade-off with one another, thus preventing any one genotype from outcompeting all others; and/ or (ii) indirectly through the effect of environmental heterogeneity enhancing the total population size and affecting structure [44]. In this study, the SFS population was both more environmentally heterogeneous, and larger in size than either of the two small cage colonies. Thus it is not possible to infer the extent to which the higher diversity within the SFS population can be attributed to these direct and indirect effects. Further investigations in which population sizes under SFS and small cage colonies are equilibrated are needed to disentangle these hypotheses. Regardless of the mechanism, it is clear that the genetic diversity of mosquito vector populations is retained at a higher degree through time in a large semi-field than small cage colonies. As reported in other studies, cage colonies had higher amount of sugars/glycogen and lower lipids amounts than SFS which may be due to the fact that, they are fed with sugar solutions as opposed to SFS which feed on plant juices [23].

These findings have particular relevance to the development of new mosquito control approaches and as an intermediate ground for testing various other new disease interventions prior to their field evaluations [54,55]. Whilst numerous studies have demonstrated that GM mosquitoes can resist infection [56] and induce sterility [57,58] under laboratory settings, previous trials using chemo-sterilized mosquitoes [59] indicate that males that perform well in the laboratory may have extremely poor fitness when released into the wild [22,33,60]. Additionally, scientists and the public have raised understandable concerns about the potential risk of unanticipated, detrimental ecological or epidemiological impacts of GM mosquitoes if they are released without a detailed understanding of how they will function in the environment [61,62]. The use of contained, semi-field systems presents an excellent opportunity to progress research on what could ultimately be an effective new disease control strategy, whilst allowing detailed study of their ecological feasibility and potential risks under contained and environmentally realistic conditions. Previously it has been demonstrated that large-scale SFS which mimic realistic environmental conditions of an African malaria transmission system can be produced [30,31,63] and that mosquito vectors can be maintained within over multiple generations that exhibit a range of realistic life-history and behavioural traits [36]. This study further strengthens the growing evidence base that SFS can provide environmentally realistic yet contained testing grounds for GM mosquitoes, by demonstrating that mosquito vectors maintained with them retain a high degree of genetic diversity, and are much more phenotypically similar to natural populations than those maintained in typical small-cage colonies in the laboratory.

Whilst, as hypothesized on the basis of these results and previous studies [30,36] that biological inferences made from SFS populations will be more realistic than those gained from research on small cage laboratory colonies, it is cautioned that there may remain other important ecological, physiological and genetic characteristics of natural mosquito vector populations that cannot fully be represented or incorporated within contained semi-field systems. For example these systems may not incorporate the full range or magnitude of mortality risks faced by mosquitoes in their natural environment, including the same diversity and abundance of predators and pathogens, predation or exposure to insecticides, pollutant and extreme weather events such as flooding and drought from which the SFS protect them from. Additionally, the constant provision of hosts and larval sites within SFS make these environments much more benign than natural settings. This environmental buffering may lead to an overestimation of mosquito fitness traits, and also could misrepresent important life-history traits. For example, several species including the marine isopod *Idotea balthica* have been observed to change their life history strategy in response to changes in predation risks [64]. Similarly, mosquito vector life history and demographic traits could be misrepresented in SFS studies if they are unable to incorporate the full range of food web dynamics in which mosquitoes are embedded. Consequently while advocating SFS as valuable, experimentally tractable systems for study of mosquito vectors under relatively realistic environmental conditions, it is cautioned that they are not a substitute for field studies. In developing this research approach, it is recommended that further comparative studies of mosquito vector fitness and ecology under SFS, field and laboratory conditions are conducted to fully highlight the benefits and limitations inherent within this method. More studies are to be expanded for female mosquitoes since the results presented in this study were on male mosquitoes, to demonstrate if the advantages of the SFS are retained for this sex, as usually it is females that are needed for study of vector ecology and control.

## Conclusions

This study presents the first comparative analysis of the genetic and phenotypic diversity of malaria vector populations colonized under traditional small-cage conditions and within a large semi-field cage. Across the first 10 generations of colonization, it is concluded that *An. arabiensis* mosquitoes reared within a large semi-field system retain a higher degree of genetic diversity through time and are much more phenotypically closer to their wild founder population. The greater similarity of individuals in the semi-field colony to their wild counterparts is hypothesized to be a product of the more realistic environmental heterogeneity that mosquitoes experience within an SFS, and the higher population sizes that these systems can maintain relative to small cage colonies. It is recommended that the SFS approach should be prioritized for the colonization of insect vector species designated for research purposes and as an intermediary testing ground for male mosquitoes destined for release in sterile and/or transgenic disease vector control programmes.
